# Polyglycolic acid sheet covering to prevent recurrence after surgery for spontaneous pneumothorax: a meta-analysis

**DOI:** 10.1038/s41598-021-83103-5

**Published:** 2021-02-09

**Authors:** Yuka Kadomatsu, Takayuki Fukui, Shoichi Mori, Toyofumi Fengshi Chen-Yoshikawa, Kenji Wakai

**Affiliations:** 1 Department of Preventive Medicine, Nagoya University Graduate School of Medicine, 65 Tsurumai-cho, Showa-ku, Nagoya, 466-8550 Japan; 2 Department of Thoracic Surgery, Nagoya University Graduate School of Medicine, Nagoya, Japan; 3 Department of Thoracic Surgery, Japanese Red Cross Nagoya Daiichi Hospital, Nagoya, Japan

**Keywords:** Diseases, Respiratory tract diseases

## Abstract

The coverage technique using absorbable mesh was first described in a European guideline published in 2015 as a preventive method for the recurrence of spontaneous pneumothorax. We performed a meta-analysis based on a literature search of primary studies that compared the postoperative recurrence rate of primary spontaneous pneumothorax between the use and nonuse of polyglycolic acid sheet coverage. Two reviewers independently selected and evaluated the quality of the relevant studies. The risk ratio in each study was calculated in a random-effect meta-analysis. Statistical heterogeneity among the included studies was quantitatively evaluated using the *I*^*2*^ index, and publication bias was assessed using a funnel plot. A total of 19 retrospective cohort studies were analyzed: 1524 patients who underwent wedge resection alone (the control group) and 1579 who received additional sheet coverage. Polyglycolic acid sheet coverage was associated with a lower recurrence rate than that in the control group (risk ratio: 0.27, 95% confidence interval 0.20–0.37, P < 0.001; *I*^2^ 0%). The funnel plot suggested possible publication bias. The covering technique reduced the recurrence rate of pneumothorax after thoracoscopic surgery to one-fourth.

## Introduction

Spontaneous pneumothorax (SP) is one of the most common respiratory conditions. Surgical excision of the underlying pathologic cause remains the procedure with the lowest recurrence rate of approximately 1% and has been recommended for cases with persistent air leakage or recurrent pneumothorax^1^. However, since the spread of thoracoscopic surgery in the 1990s, SP’s high recurrence rate has become a concern^2^ likely because the risk for recurrence was reported to be four-fold increased after thoracoscopic surgery compared with thoracotomy^3^. Therefore, reducing the SP recurrence rate after thoracoscopic surgery is an urgent task for thoracic surgeons.

Several preventive procedures are available during thoracoscopic surgery, including pleurectomy, chemical pleurodesis, mechanical abrasion, and staple line covering^4^. Two large meta-analyses reported that chemical pleurodesis had the lowest recurrence rate of 1.7% and was associated with a significant reduction of recurrence by 69% compared with the recurrence rate after wedge resection alone; however, both meta-analyses only included a few trials assessing methods of coverage (7 of 51 and in 1 of 29, respectively)^5,6^. In addition, most studies included in the previous meta-analysis added other procedures in addition to coverage, such as abrasion and pleurodesis.

In terms of guidelines, in the most recent European Respiratory Society (ERS) statement on primary SP, coverage of the stapling line with absorbable mesh was first introduced^7^. In the statement, they described that coverage technique seemed to improve decreasing SP recurrence, but they also suggested that it should be confirmed in further studies^7^.

Staple line coverage has been mainly used in Asian countries, such as China, Korea, and Japan^8–11^. Despite the widespread use of staple line coverage in clinical practice, most previous studies that assessed the effect of coverage were small case series, including studies from a single-center experience or in a setting without a control group. In the present meta-analysis, to avoid high heterogeneity among the included studies, we only focused on polyglycolic acid (PGA) materials and excluded studies that involved an additional procedure other than staple line coverage, such as pleurodesis or pleurectomy.

We aimed to systematically assess the efficacy of covering the staple line with a PGA sheet to prevent SP recurrence after thoracoscopic surgery. This study excludes studies that added major techniques other than coverage and is the first meta-analysis to examine the pure preventive effect of coverage on the recurrence of pneumothorax.

## Results

### Literature search and quality assessment

**Figure 1:**
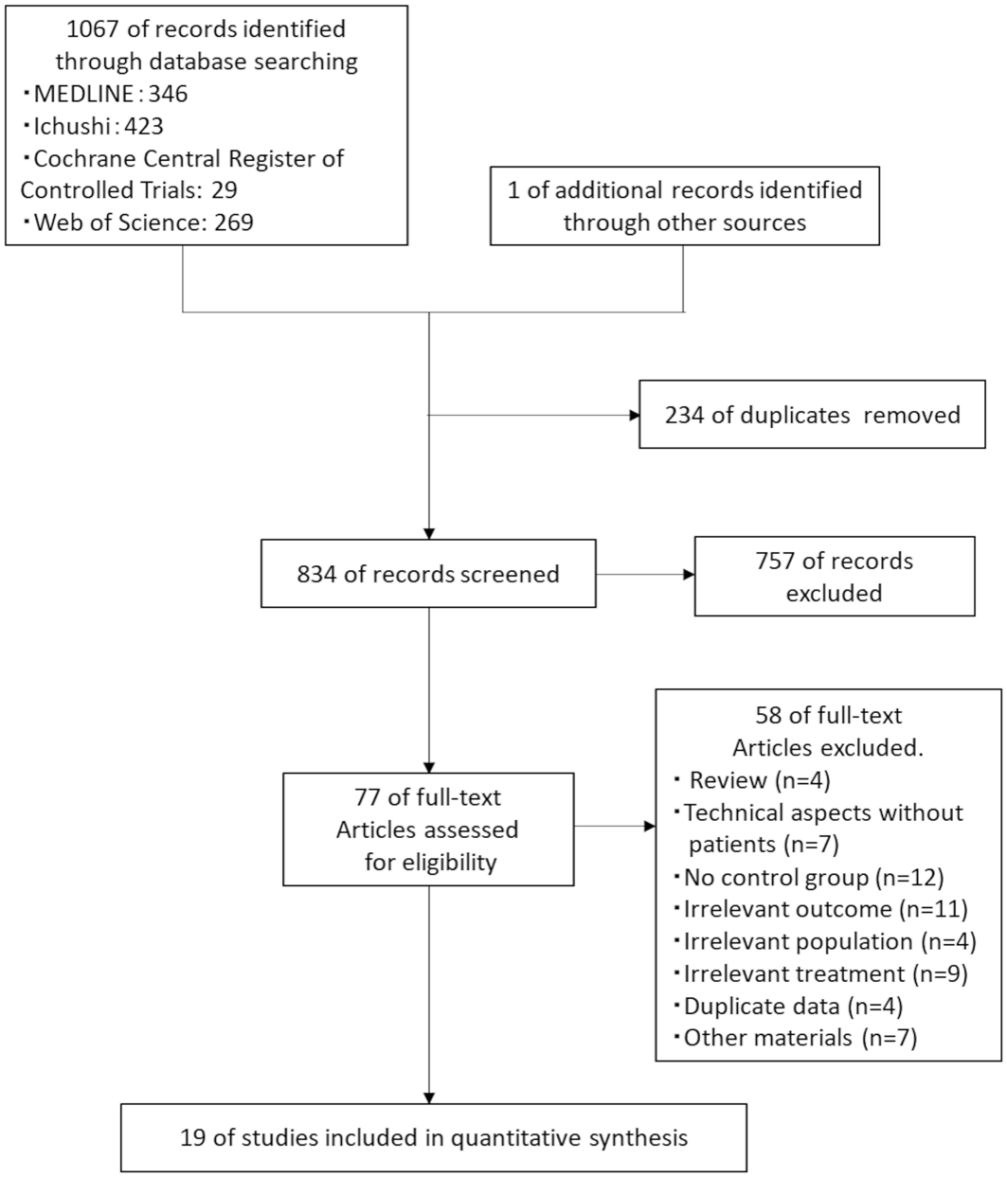
PRISMA flowchart for clinical outcomes^41^. *PRISMA* preferred reporting items for systematic reviews and meta-analyses.

We identified 1067 studies using the search strategy and one additional article from a manual search of the included references (Fig. 1). After applying the inclusion and exclusion criteria, 19 studies remained eligible for analysis^12–30^. The Newcastle–Ottawa Scale assessments for these studies are presented in Supplementary Table E1; for the eight recommended items, the mean number of stars awarded was 4.6 (range, 3–7).

### Study details

The characteristics of the included studies were summarized in Table 1. We contacted the first author of one article that did not contain the cumulative incidence and received data on the recurrence rate in each group by e-mail. All the studies were retrospective cohort studies conducted in Japan; there was no eligible study from other countries. The 19 studies comprised 1579 patients who underwent wedge resection plus staple line coverage or buttress stapling with PGA sleeve (PGA covering group) and 1524 who underwent wedge resection alone (control group). Seventeen studies using the PGA sheet added a patch after stapling, and the other two studies used the PGA sleeve at the time of stapling^15,23^. In 11 studies, the control and PGA covering groups were sequentially studied (sequential group)^12,13,17,19,22–27,30^, whereas the other 8 studies compared the control and PGA covering groups during the same study period (concurrent group)^14–16,18,20,21,28,29^. Ishida et al. reported the recurrence rates for three covering technique subgroups, namely, PGA only, PGA + autologous blood, and PGA + fibrinogen^21^. Six studies included only subjects with primary SP^13,17,23,26,27,29^. In one study, approximately 22% of the subjects had secondary pneumothorax^20^, and 12 studies did not describe the pneumothorax cause^12,14–16,18,19,21,22,24,25,28,30^. Regarding postoperative complications other than air leakage, Urabe et al. reported three cases of high fever after discharge, whereas two studies reported no major complications in the control and PGA covering groups^15,23,24^.

**Table1:** Characteristics of the included studies.

Author	Year	No. of patients, PGA group	No. of patients, control group	Method	Intervention	Analysis	Period	Inclusion criteria	Additional inclusion criteria^d^	Age (years)	Follow-up period (months)	
											PGA group	Control group
Yoshihara	1999	62	116	VATS	PGA sheet + fibrinogen	Sequential	1994–1997–1999	SP	Initial surgery in under 40 years of age	24.6^a^	12.5^b^	13.4^b^
Nakanishi	2001	41	38	VATS	PGA sheet + fibrinogen	Sequential	1996–1998–2000	Primary SP		32.5^a^	11.2 ± 5.8^b^	43.5 ± 16.1^b^
Minami	2003	19	30	VATS	PGA (sleeve)	Concurrent	1999–2002	SP	Initial surgery	32.5^a^	10.8^b^	
Noda	2003	35	19	VATS	PGA sheet + fibrinogen	Concurrent	1996–2001	SP	Male patients of under 61 years of age	ND	24	
Yamagata	2004	101	33	VATS	PGA sheet + fibrinogen	Concurrent	1989–2003	SP	Initial surgery	31.1^a^	ND	
Matsukura	2004	124	167	VATS	PGA sheet	Sequential	1994–1999–2004	Primary SP		ND	10.5^b^	16.1^b^
Noda	2005	99	15	VATS	PGA sheet + fibrinogen	Concurrent	2002–2003	SP		31 ± 17.5^b^	ND	
Ichinari	2007	32	26	VATS	PGA sheet + autologous blood	Sequential	1995–2001–2005	SP		ND	25.4^c^	52.6^c^
Ueshima	2007	11	62	VATS	PGA sheet	Concurrent	1994–2004	Primary and secondary SP		33.1^b^	ND	
Ishida	2007	42	58	VATS	PGA sheet ± fibrinogen or autologous blood	Concurrent	2004–2006	SP	Initial surgery in under 40 years of age	22.4^a^	10 ± 6^b^	24 ± 8^b^
Nogimura	2008	16	17	VATS, Minithoracotomy	PGA sheet	Sequential	2001–2003–2006	SP	Initial surgery in under 26 years of age	18^c^	ND	
Urabe	2008	210	164	VATS	PGA (sleeve)	Sequential	1995–2000–2005	Primary SP		25.4^b^	ND	
Tajima	2009	56	32	VATS	PGA sheet	Sequential	2002–2004–2006	SP	Initial surgery in under 26 years of age	20^a^	33 ± 13^b^	25 ± 7^b^
Asakura	2011	86	121	VATS	PGA sheet	Sequential	1995–2005–2011	SP		ND	ND	
Hirai	2015	181	98	VATS, Thoracotomy	PGA sheet + fibrinogen	Sequential	1994–2002–2007	Primary SP		29.4^a^	54.6 ± 17.2^b^	131.1 ± 52.4^b^
Inafuku	2016	108	44	VATS	PGA sheet + fibrinogen	Sequential	2001–2007–2010	Primary SP	Initial surgery	24.7^b^	21.1^b^	
Kimura	2017	32	33	VATS	PGA sheet + platelet-rich plasma	Concurrent	2008–2011	SP		27.7^a^	18	
Nakayama	2017	105	62	VATS	PGA sheet + fibrinogen	Concurrent	2005–2015	Primary SP	Surgery in under 41 years of age	23^c^	4	
Miyahara	2017	219	389	VATS	PGA sheet ± fibrinogen	Sequential	1993–2007–2015	SP	Initial surgery	38^b^	ND	

*PGA* polyglycolic acid, *SP* spontaneous pneumothorax, *VATS* video-assisted thoracoscopic surgery, *ND* neither described nor discernible from the original manuscript.

^a^Values calculated from mean age of each group.

^b^Values of mean, or mean ± standard deviation.

^c^Values of median.

^d^Every study examined the patients who underwent surgery for spontaneous pneumothorax. This column shows the additional inclusion criteria that each study set for inclusion.

### Preventive effect of the PGA sheet

**Figure 2:**
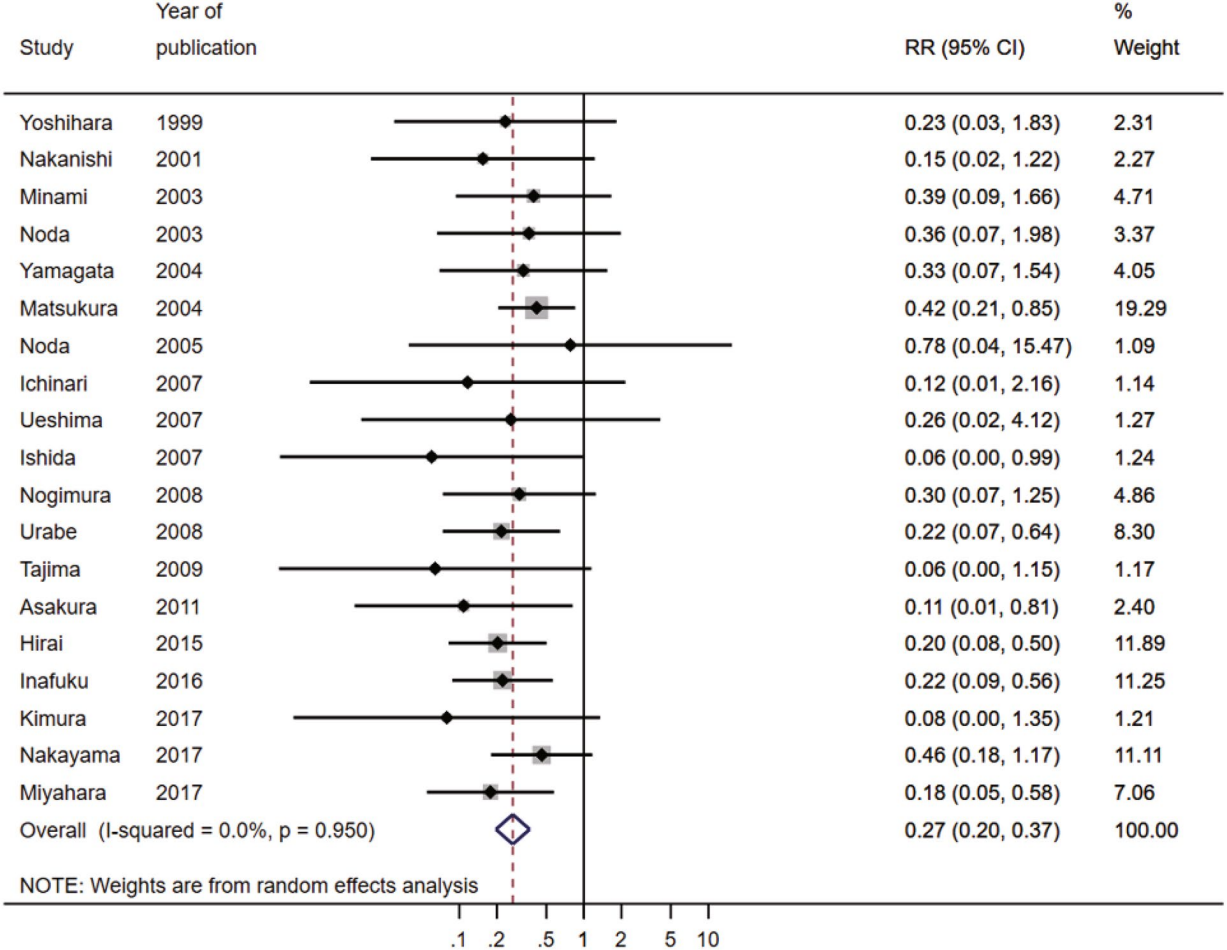
Forest plot of the risk ratio for recurrence. *CI* confidence interval, *RR* risk ratio.

The pooled risk ratio (RR) showed that the recurrence rate was significantly lower in the PGA-covered group compared with the control group (RR 0.27, 95% confidence intervals (CI) 0.20–0.37, P < 0.001, *I*^2^ 0%; Fig. 2). The sensitivity analysis showed that the overall effect was not influenced by omitting a study that included patients with secondary SP ^20^ and two studies that adapted the PGA sleeve^15,23^. Analysis using a fixed effects model revealed a similar pooled result (RR 0.24, 95% CI 0.18–0.33, P < 0.001, *I*^2^ 0%).

### Publication bias

**Figure 3:**
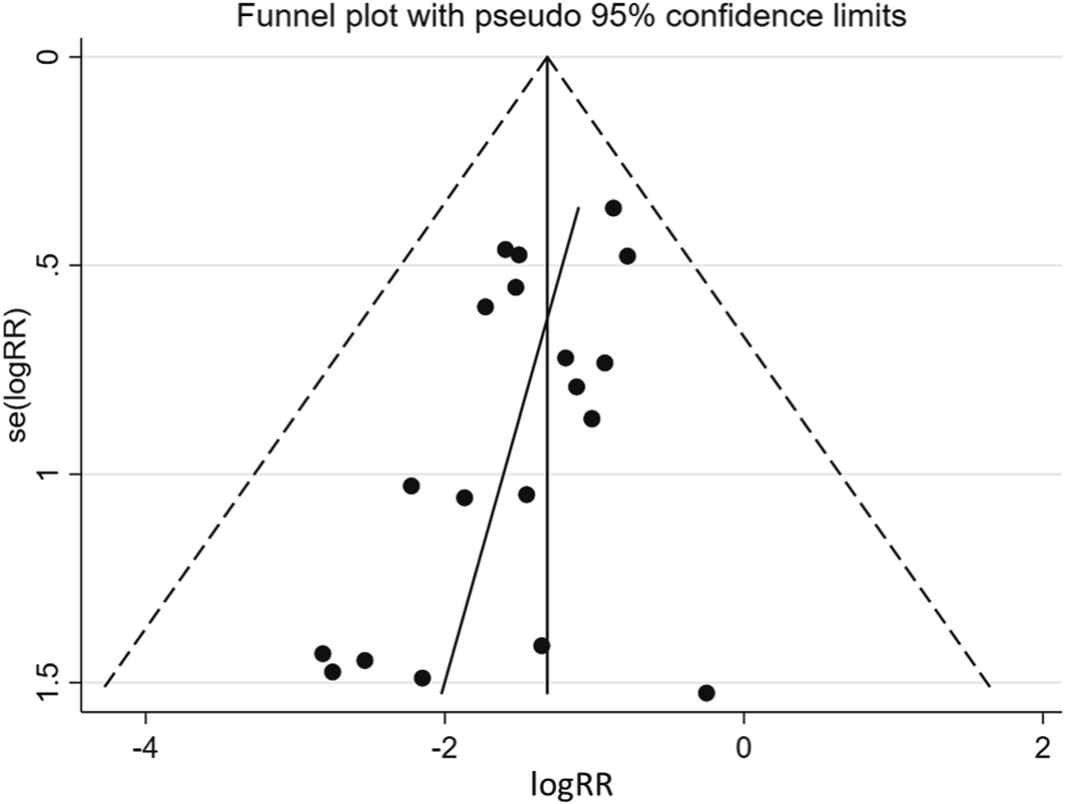
Funnel plot of the included studies. The asymmetrical plot indicates publication bias. *RR* risk ratio.

The funnel plot used to assess publication bias was shown in Fig. 3. The regression line tilted up slightly to the right, and a sparse area was noted in the lower right field. Egger’s test for bias yielded a P-value of 0.034, which suggested publication bias to a lower summary RR. The trim-and-fill method did not detect publication bias, was not applied to any of the studies, and did not change the pooled result (RR 0.27, 95% CI 0.20–0.37, P < 0.001).

### Subgroup analyses

**Figure 4:**
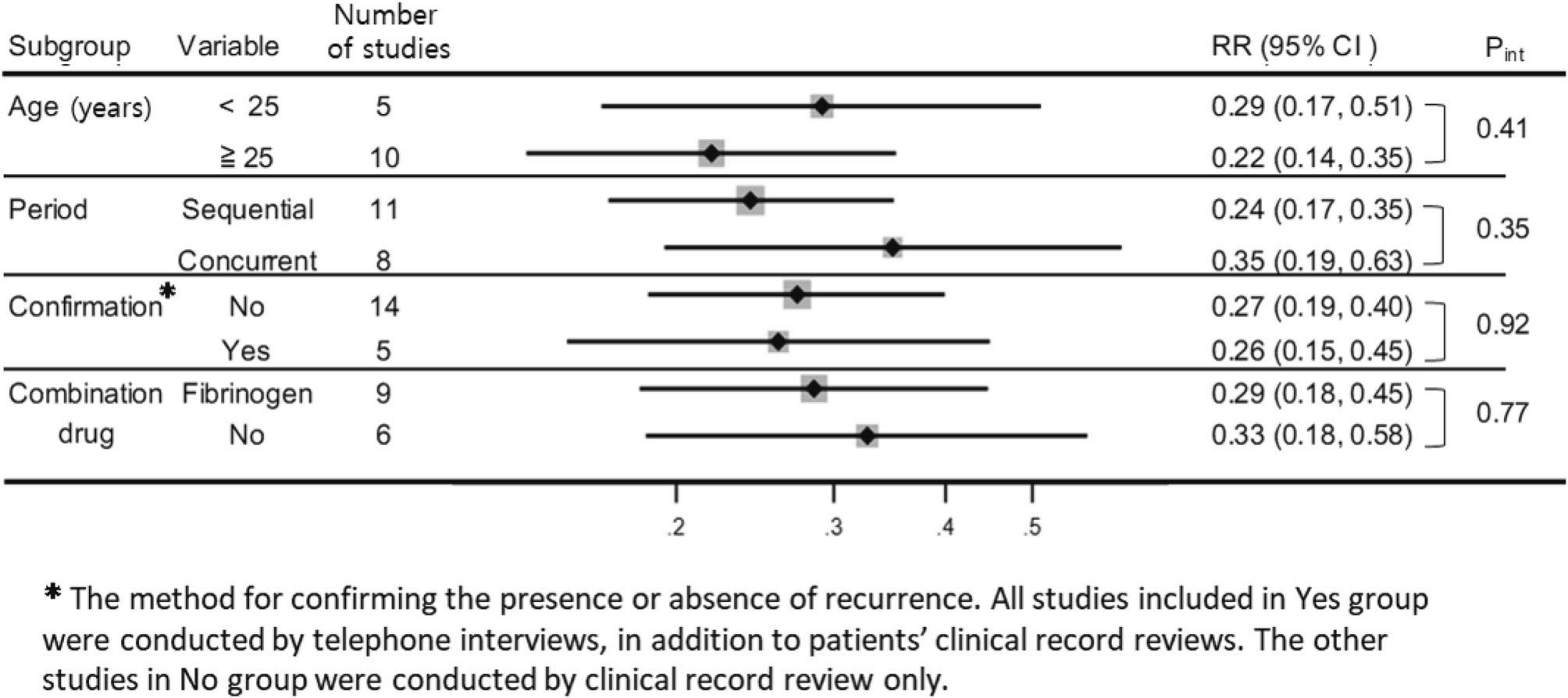
Subgroup analysis of the effect of the PGA covering. *CI* confidence interval, *RR* risk ratio, *Pint* P for interaction.

The subgroup analysis results for age category, combination drug, method of data collection (sequential or concurrent), and strategy for confirming recurrence were shown in Fig. 4. A significantly smaller RR than unity was noted in all subgroups with a lower recurrence rate in the PGA-covered group than in the control group. The zero *I*^*2*^ statistics for each subgroup indicated the absence of heterogeneity in any subgroup set.

## Discussion

This study investigated the preventive effect of the PGA covering technique on SP postoperative recurrence. The pooled effect size suggested that the PGA covering technique reduced postoperative SP recurrence to one-fourth. In this meta-analysis, two types of coverage techniques were assessed. One was PGA sheet addition of a patch after stapling, and the other was PGA sleeve reinforcement incorporated with a stapling device. The number of studies was insufficient for subgroup analysis to compare the RR of each technique, but the pooled RR did not change by omitting PGA sleeve studies.

Postoperative SP recurrence was thought to be caused by overlooked and/or regenerated bullae, which mainly develop adjacent to the staple lines^26,31^. PGA coverage of the staple line can relieve the tension forces arising from the use of staplers and thicken the visceral pleura to prevent rupture of a regenerated bulla. A PGA sheet is an absorbable reinforcement material that is metabolized in approximately 15 weeks^32^. The reported formation of granulation and inflammatory reactions during absorption suggested that the PGA sheet helped prevent recurrence^33^. However, based on a study on chest computed tomography images acquired 1 year after surgery, Onuki et al. reported that although the PGA covering reduced the SP recurrence rate, it did not prevent neogenetic bullae occurrence^31^. In a study comparing absorbable mesh covering with mechanical pleurodesis, Cho et al. reported no difference in the frequency of new bullae; however, the PGA covering group exhibited a slightly reduced frequency of bullae occurrence at the staple line compared with mechanical pleurodesis^34^. Ten studies included in the present meta-analysis discussed the cause of recurrence^12,13,15,17,18,23,24,30,31^; of these, seven concluded that the area along the staple line was not a cause of postoperative recurrence^12,13,15,18,24,30,31^. This meta-analysis excluded studies that adopted other major additional procedures to examine the impact of genuine coverage techniques. However, due to the local effect of the coverage technique, the “recurrence” may have contained not only the initial treatment site recurrence but also the new ipsilateral pneumothorax caused by the bullae formation apart from the initial treatment site, which has the possibility of weakening the effect of the coverage technique. Further studies are needed to clarify the biological mechanisms of degradable sheet coverings in preventing recurrence and bullae genesis.

Urabe et al. reported that 3 of 201 cases (1.5%) in their PGA-covered group experienced persistent fever after discharge, but this symptom was controlled by the administration of anti-inflammatory analgesics^23^. Conversely, two other studies reported no severe complications, such as allergic reactions or infections^15,24^. Given that surgical invasion itself can cause postoperative fever, even in a normal clinical course, cases of persistent fever resulting from PGA coverage may have been overlooked. Nakamura et al. reported that the incidence of infections following pulmonary resection did not differ according to the use of PGA^35^. Although data on the safety of PGA coverage are scarce, no mortality or fatal comorbidity was reported by any of the studies included in our analysis.

We conducted several subgroup analyses for the main outcome. The beneficial effect of PGA coverage seemed to be greater in the older population group compared with the younger group and in the sequential PGA coverage group compared with the concurrent PGA coverage group. However, the differences were not statistically significant. Younger age has been reported to be a risk factor for pneumothorax recurrence^29,36,37^. Our subgroup analysis suggested that younger patients experience less benefit from additional treatments. The greater effect observed in the sequential studies compared with the concurrent studies on PGA coverage may have been due to improvements in the surgical procedural devices. Furthermore, the group studied earlier, i.e., the control group, was inevitably observed for a relatively long follow-up period; this phenomenon might have resulted in more cases of recurrence being observed in this group compared with the recurrent cases in the PGA covering group.

In the present meta-analysis, the most combined drug with PGA covering was fibrin glue, which is generally a well-tolerated biological material. However, concerns include the risks for immunologic reaction to the sealants (2%), excessive or uncontrolled clotting, and transmission of some viral pathogens^38,39^.

This study had several limitations. First, all the studies included in the analysis were not randomized trials and eliminating the potential effects of confounding factors was impossible. These features weakened the evidence. Second, although we did not impose any restriction on the country where the studies were conducted, all the included studies were from Japan. Therefore, our results may not apply to patients of other ethnicities. Third, the follow-up period might have been insufficient or unreported in some studies. In general, there has been no consensus on the feasible duration of the follow-up period after surgery. An inadequate follow-up period may have resulted in missed cases of recurrence and an underestimated recurrence rate. However, our main index was comparing the RR for recurrence between groups, and the absolute recurrence rates were not discussed. Therefore, this limitation should not have a substantial impact on our main finding. Fourth, our main target was primary SP, but we were unable to completely separate primary and secondary SP cases given the lack of information in the included studies. However, considering that the reported peak age of secondary SP was approximately 60 years^40^ and that the participants’ average age in most of the studies in this meta-analysis was less than 35 years, the contamination’s impact was not large enough to reverse our main result. Finally, the asymmetrical distribution of studies in the funnel plot may have been due to unpublished smaller studies that showed no statistically significant effects. To avoid further publication bias, the findings of the present meta-analysis need careful consideration.

## Methods

### Search strategy

This study was designed, performed, and reported following the Preferred Reporting Items for Systematic Reviews and Meta-Analyses guidelines^41^. The protocol was registered in PROSPERO (CRD42019129157). A preliminary search identified eligible reports and estimated the volume of literature on the topic. The search strategy was applied using Online Databases (PubMed/MEDLINE, Web of Science, Cochrane Central Register of Controlled Trials, Ichushi; the database of medical literature published in Japan). We included a Japanese database based on a report that 7637 of 11,835 patients (64.5%) who underwent surgical operations for SP in 2016 and received staple line coverage in Japan^42^. Search strategies (see Supplementary Material) were constructed for MEDLINE and Ichushi. The MEDLINE strategy was adapted for the Cochrane Central Register of Controlled Trials and Web of Science. The search was conducted for studies published since the databases’ inceptions until May 31, 2019, applying English and Japanese language filters. In addition, the reference lists of the retrieved studies were manually reviewed to search for further reports for inclusion.

### Selection criteria

The primary outcome was SP recurrence. We assessed the impact of PGA coverage on preventing recurrence by comparing it to SP recurrence without the use of a covering technique. We then systematically reviewed primary studies that met the following inclusion criteria: investigated primary SP that was treated by wedge resection using an endoscopic stapling device followed by PGA sheet coverage or resection using buttress stapling with PGA felt; compared the effectiveness of SP management between a PGA group and a control group; and English or Japanese as the language of publication. We applied the following exclusion criteria: animal studies, involvement of other additional procedures (such as chemical pleurodesis, pleurectomy, and mechanical abrasion), lack of a control group, use of other covering materials (such as polyglactin mesh or oxidized cellulose mesh), inclusion of several covering materials (including PGA) into a single group, the main targeted population including patients with pneumothorax secondary to a preexisting lung disease or trauma or an iatrogenic cause, and insufficient data on the agents or techniques used. Abstracts, editorials, case reports, case series, systematic reviews, meta-analyses, and unpublished articles were also excluded. When the studies had overlapping data, we selected the one with the latest and most extensive dataset. Two researchers (Y.K. and T.F.) assessed the studies for inclusion using the abovementioned criteria. Any disagreement was carefully resolved through discussion among the researchers, including a third author (K.W.) if necessary.

### Study selection, data extraction, and risk of bias assessment

Two authors (Y.K. and T.F.) independently reviewed all the articles identified in the search. After full document screening of the relevant articles, each author independently extracted the data. The primary index was the RR for pneumothorax recurrence. The data extracted from each study included the year of publication, authors’ names, country, study design, sample size, cumulative incidence of events, age and sex of the subjects, combination of drugs (fibrinogen, autologous blood, etc.), duration of follow-up, adverse events, and method for confirming the presence or absence of recurrence. If the primary outcome was not reported as the cumulative incidence, we asked the authors of that particular study for information via e-mail. During data extraction, the quality of the studies was assessed according to the Newcastle–Ottawa Scale^43^. In case of any disagreement between the two authors, the opinion of the third author (K.W.) was sought, and the matter was resolved by consensus.

### Statistical analysis

The RR in each study was calculated from the cumulative incidence of pneumothorax recurrence during the study period in the PGA and control groups. The RRs were synthesized in a random-effect meta-analysis and into a pooled estimate based on the log (RR) estimates and the corresponding 95% CIs using the method of DerSimonian and Laird^44^. If a study reported zero recurrences in the PGA or control group, a standard correction of 0.5 was added to the sample size and to the number of events in each group to avoid computational errors. Subgroup analyses were performed based on age category, type of treatment, data collection method (sequential or concurrent), and method used to confirm recurrence. In the subgroup analyses of age, when a report provided only the median value without the mean value, the median was used in place of the mean. When the mean age of all subjects in a study was not provided, it was calculated from the mean age of the groups. A study with a missing value was excluded from the corresponding subgroup analyses. A sensitivity analysis was performed by omitting the studies that might increase heterogeneity for each outcome to determine how certain factors might influence the overall effects of each factor. Heterogeneity among the included studies was evaluated using the *I*^2^ statistic^45^. Graphic representation of any potential publication bias was generated by a funnel plot of the natural logarithms of the RRs vs. their standard errors; it was assessed visually by applying Egger’s test for publication bias^46,47^. The trim-and-fill method was adopted to adjust for any publication bias that was detected^48^. Statistical analyses were performed using Stata with metan and metafunnel commands (ver. 15.1; Stata Corporation, College Station, TX, USA). All P-values were two-sided, and P-values < 0.05 were considered statistically significant.

## Conclusion

To the best of our knowledge, this study was the first meta-analysis to examine the pure preventive effect of PGA coverage during thoracoscopic surgery on a large scale and compared with the effects of wedge resection alone. The results showed that PGA coverage could help prevent pneumothorax recurrence. Although interpreting these results would require careful consideration, this meta-analysis has provided the largest-scale data that can support the PGA covering method’s effect on preventing SP recurrence. Since PGA coverage is a simple and possibly effective method, we hope that clinical trials on this method will be conducted on a larger population.

## Supplementary Information

Supplementary Information 1.

Supplementary Information 2.
