# Supplementary material for: Polyglycolic acid sheet covering to prevent recurrence after surgery for spontaneous pneumothorax: a meta-analysis
Source: Sci Rep. 2021 Feb 9;11:3392. doi: 10.1038/s41598-021-83103-5 (PMC7873206; doi:10.1038/s41598-021-83103-5)
Supplement: Supplementary file 2 — Supplementary Information 2. [file 41598_2021_83103_MOESM2_ESM.pdf]

Polyglycolic Acid Sheet Covering to Prevent Recurrence After Surgery for Spontaneous Pneumothorax: A Meta-analysis

Authors: Yuka Kadomatsu[1, 2, *], Takayuki Fukui[2], Shoichi Mori[3], Toyofumi Fengshi Chen-Yoshikawa[2], Kenji Wakai[1]

[1]Department of Preventive Medicine, Nagoya University Graduate School of Medicine, Nagoya, Japan
[2]Department of Thoracic Surgery, Nagoya University Graduate School of Medicine, Nagoya, Japan
[3]Department of Thoracic Surgery, Japanese Red Cross Nagoya Daiichi Hospital, Nagoya, Japan

[*]**Corresponding Author**:
Yuka Kadomatsu
Address: 65 Tsurumai-cho, Showa-ku, Nagoya 466-8550, Japan
Telephone No.: +81-52-744-2375
Fax No.: +81-52-744-2971
E-mail address: yuka.kadomatsu@gmail.com

Supplementary Material E1: Search strategies for MEDLINE and Ichishi

A. MEDLINE (EBSCO)

1. [TX] cover*
2. [TX] reinforce*
3. [TX] PGA
4. [TX] polyglycolic acid
5. [TX] biodegrade*
6. [TX] absorb*
7. [TX] staple*
8. 1 OR 2 OR 3 OR 4 OR 5 OR 6 OR 7
9. [MH] "Pneumothorax" OR [TX] pneumothorax
10. [TX] recurren*
11. [TX] thoracoscop*
12. [TX] surger*
13. [TX] bullect*
14. [TX] operati*
15. 10 OR 11 OR 12 OR 13 OR 14
16. 8 AND 9 AND 15
17. 15/Filters: Language: -english

B. Ichushi (医中誌)
1. [気胸]/TH
2. ("コーティング(薬学)" /TH or 被覆/AL)
3. 補強/AL
4. ("Polyglycolic Acid" /TH or PGA/AL)
5. メッシュ/AL
6. (再発/TH or 再発/AL)
7. (外科手術/TH or 手術/AL)
8. 嚢胞切除/AL
9. (ビデオ胸腔鏡手術/TH or VATS/AL)
10. #2 or #3 or #4 or #5 or #6
11. #7 or #8 or #9
12. #1 and #10 and #11
13. (#12) and (PT = 会議録除く)
14. (#13) and (LA = 日本語 (PT = 症例報告・事例除く) AND (PT = 原著論文, 会議録除く))
15. (#13) and (LA = 英語 (PT = 症例報告・事例除く) AND (PT = 原著論文, 会議録除く))

Table E1:　Quality assessment of studies in the meta-analysis based on Newcastle-Ottawa Scale for Cohort Studies

			Selection				Comparability	Outcome			
	Author	Year	Representativeness of exposed cohort (Max：★)	Selection of non-exposed cohort (Max：★)	Ascertainment of exposure (Max：★)	Demonstration that outcome of interest was not present at start of study (Max：★)	Comparability of cohorts on the basis of the design or analysis (Max：★★)	Assessment of outcomes (Max：★)	Was follow-up long enough for outcomes to occur (Max：★)	Adequacy of follow up of cohorts (Max：★)	Total score (out of 9)
1	Yoshihara	1999	★	★	★	N/A	―	★	★	―	5
2	Nakanishi	2001	★	★	★	N/A	―	★	―	―	4
3	Minami	2003	★	★	★	N/A	★	★	―	―	5
4	Noda	2003	―	★	★	N/A	―	★	★	―	4
5	Yamagata	2004	★	★	★	N/A	★	★	―	―	5
6	Matsukura	2004	★	★	★	N/A	―	★	―	―	4
7	Noda	2005	★	★	★	N/A	―	★	―	―	4
8	Ichinari	2007	★	★	★	N/A	―	★	★	―	5
9	Ueshima	2007	―	★	★	N/A	―	★	―	―	3
10	Ishida	2007	★	★	★	N/A	―	★	―	―	4
11	Nogimura	2008	―	★	★	N/A	―	★	―	―	3
12	Urabe	2008	★	★	★	N/A	―	★	★	★	6
13	Tajima	2009	―	★	★	N/A	―	★	★	★	5
14	Asakura	2011	★	★	★	N/A	―	★	―	―	4
15	Hirai	2015	★	★	★	N/A	―	★	★	―	5
16	Inafuku	2016	★	★	★	N/A	―	★	★	★	6
17	Kimura	2016	★	★	★	N/A	★	★	★	★	7
18	Nakayama	2017	★	★	★	N/A	―	★	―	★	5
19	Miyahara	2017	★	★	★	N/A	―	★	―	―	4
